# Evaluation of the Bruker Biotyper Matrix-Assisted Laser Desorption/Ionization Time-of-Flight Mass Spectrometry System for Identification of Clinical and Environmental Isolates of *Burkholderia pseudomallei*

**DOI:** 10.3389/fmicb.2016.00415

**Published:** 2016-04-08

**Authors:** He Wang, Ya-Lei Chen, Shih-Hua Teng, Zhi-Peng Xu, Ying-Chun Xu, Po-Ren Hsueh

**Affiliations:** ^1^Department of Clinical Laboratory, Peking Union Medical College Hospital, Chinese Academy of Medical SciencesBeijing, China; ^2^Department of Biotechnology, National Kaohsiung Normal UniversityKaohsiung, Taiwan; ^3^Department of Graduate Institute of Biomedical Sciences, Chang Gung UniversityTao-Yuan, Taiwan; ^4^Departments of Laboratory Medicine and Internal Medicine, National Taiwan University Hospital, National Taiwan University College of MedicineTaipei, Taiwan

**Keywords:** *Burkholderia pseudomallei*, *B. thailandensis*, matrix-assisted, laser desorption/ionization time-of-flight mass spectrometry, 16S rDNA gene sequencing analysis, enhanced database

## Abstract

*Burkholderia pseudomallei* is not represented in the current version of Bruker Biotyper matrix-assisted laser desorption/ionization time-of-flight mass spectrometry (MALDI-TOF MS) system. A total of 66 isolates of *B. pseudomallei*, including 30 clinical isolates collected from National Taiwan University Hospital (NTUH, *n* = 27) and Peking Union Medical College Hospital (PUMCH, *n* = 3), and 36 isolates of genetically confirmed strains, including 13 from clinical samples and 23 from environmental samples, collected from southern Taiwan were included in this study. All these isolates were identified by partial 16S rDNA gene sequencing analysis and the Bruker Biotyper MALDI-TOF MS system. Among the 30 isolates initially identified as *B. pseudomallei* by conventional identification methods, one was identified as *B. cepacia* complex (NTUH) and three were identified as *B. putida* (PUMCH) by partial 16S rDNA gene sequencing analysis and Bruker Biotyper MALDI-TOF MS system. The Bruker Biotyper MALDI-TOF MS system misidentified 62 genetically confirmed *B. pseudomallei* isolates as *B. thailandensis* or *Burkholderia* species (score values, 1.803–2.063) when the currently available database (DB 5627) was used. However, using a newly created MALDI-TOF MS database (including *B. pseudomallei* NTUH-3 strain), all isolates were correctly identified as *B. pseudomallei* (score values >2.000, 100%). An additional 60 isolates of genetically confirmed *B. cepacia* complex and *B. putida* were also evaluated by the Bruker Biotyper MALDI-TOF MS system using the newly created database and none of these isolates were identified as *B. pseudomallei*. MALDI-TOF MS is a versatile and robust tool for the rapid identification of *B. pseudomallei* using the enhanced database.

## Introduction

Melioidosis is a tropical and subtropical infectious disease caused by *Burkholderia pseudomallei*, a Gram-negative, aerobic, motile rod-shaped bacterium that is widely distributed in rice field soil, and in stagnant water throughout the tropics (Hsueh et al., 2001; Currie et al., 2008; Lau et al., 2014). *B. pseudomallei* is also a major cause of community-acquired septicemia and pneumonia in adults in the Asia-pacific region, particularly in northeast Thailand (Peto et al., 2014). In Taiwan, the first case of melioidosis was reported in 1985 in a man who acquired the disease after aspirating river water in a near-drowning accident in the Philippines (Lee et al., 1985) Since then several sporadic and epidemic cases have been reported (Hsueh et al., 2001; Ko et al., 2007; Su et al., 2011; Chen et al., 2013, 2014). Previous studies clearly showed that the disease was endemic in Taiwan and demonstrated that all clinical isolates were arabinose non-assimilators (Hsueh et al., 2001; Chen et al., 2013). Other studies have reported high concentrations of ambient *B. pseudomallei* during typhoon season in regions of Taiwan (Ko et al., 2007; Su et al., 2011; Chen et al., 2014). Several typhoon-related melioidosis epidemics have also been reported (Ko et al., 2007; Su et al., 2011). There is also evidence that melioidosis can be transmitted to humans via environmental aerosols contaminated with *B. pseudomallei* (Chen P. S. et al., 2015).

In Mainland China, melioidosis was first reported in 1990 (Yang, 2000) and is now known to be endemic to several tropical provinces, including Hainan, Guangdong, and Guangxi (Chen H. et al., 2015; Fang et al., 2015; Zheng et al., 2015). Multilocus sequence typing (MLST) disclosed that *B. pseudomallei* ST562 is dominant in southern China as well as in Australia and Taiwan and that its wide-ranging presence might be due to recent spread caused by transmission between regions (Chen H. et al., 2015). Whole-genome sequencing of *B. pseudomallei* has been conducted for isolates obtained from patients with melioidosis in Mainland China (strain BPC006) and Taiwan (strain vgh07; Fang et al., 2012; Chen Y. S. et al., 2015).

MALDI-TOF MS is increasingly being used in clinical microbiology laboratories to identify bacterial isolates to the species level and the technique is expected to further accelerate the routine identification of suspicious isolates (Bizzini and Greub, 2010; Inglis et al., 2012; Lau et al., 2012; Niyompanich et al., 2014; Jang et al., 2015; Lasch et al., 2015). Because diseases due to *B. pseudomallei* are uncommon in North America and Europe, *B. pseudomallei* are not included (but *B. thailandensis* was included) in the reference spectra of the Bruker Biotyper and Vitek MS libraries (SARAMIS database) (Jang et al., 2015).

In the present study, we evaluated the ability of the Bruker Biotyper MALDI-TOF MS system to accurately identify genetically confirmed *B. pseudomallei* that were recovered from patients and environmental sources.

## Materials and methods

### Bacterial isolates

A total of 66 isolates of *B. pseudomallei*, including 43 clinical isolates and 23 environmental isolates, were collected for study. Among these isolates, 27 were recovered from patients with bloodstream infections who were treated at National Taiwan University Hospital (NTUH, a 2500-bed university-affiliated hospital in northern Taiwan) during the period 1998–2014 (Table 1), and three isolates were recovered from patients treated at Peking Union Medical College Hospital (PUMCH), Beijing, China. The other 36 isolates of *B. pseudomallei*, which had been identified by sequencing of the 16S rRNA and flagellar genes (Chen et al., 2013), included 13 isolates from various clinical samples obtained from patients treated at several hospitals in southern Taiwan and 23 isolates obtained from different environmental sources during the period 1994–2013 (Table 2; Chen et al., 2013). Cultures and analysis of *B. pseudomallei* isolates were manipulated in the Mycobacteriology Laboratory at NTUH, a biosafety level 3 laboratory, and followed the biosafety level 3 precaution. Identification of these isolates were initially based on conventional biochemical methods and commercial identification systems, including API (API 20E) and Vitek 2 (ID-GN card) (bioMe'rieux, Marcy l'Etoile, France). Arabinose assimilation testing was performed for all genetically confirmed *B. pseudomallei* isolates as reported previously (Hsueh et al., 2001).

**Table 1:** **Results of 16S rRNA sequencing analysis and Bruker Biotyper MALDI TOF MS for the identification of 26 isolates of ***B. pseudomallei*** recovered from patients who were treated at National Taiwan University Hospital (NTUH)**.

**No. (NTUH)**	**Year of isolation**	**Identification by 16S rRNA sequencing analysis**		**Identification by Bruker Biotyper MALDI TOF MS system**			
		**Species (% of identity)**	**Accession no (best match)**.	**Database (DB 5627)**		**New database (NTUH-3 strain)**	
				**Organism (best match)**	**Score value**	**Organism (best match)**	**Score value**
1.	1998	*B. pseudomallei* (100)	CP004043.1	*B. thailandensis*	2.063	*B. pseudomallei*	2.15
2.	1998	*B. pseudomallei* (100)	CP004043.1	*B. thailandensis*	1.913	*B. pseudomallei*	2.228
3.	1998	*B. pseudomallei* (100)	CP004043.1	*B. thailandensis*	2.036	*B. pseudomallei*	2.318
4.	1999	*B. pseudomallei* (100)	CP004043.1	*B. thailandensis*	1.974	*B. pseudomallei*	2.257
5.	1999	*B. pseudomallei* (100)	CP004043.1	*B. thailandensis*	1.993	*B. pseudomallei*	2.332
6.	2000	*B. pseudomallei* (100)	CP004043.1	*B. thailandensis*	1.907	*B. pseudomallei*	2.161
7.	2000	*B. pseudomallei* (100)	CP004043.1	*B. thailandensis*	1.907	*B. pseudomallei*	2.117
8.	2000	*B. pseudomallei* (100)	CP004043.1	*B. thailandensis*	1.899	*B. pseudomallei*	2.266
9.	2000	*B. pseudomallei* (100)	CP004043.1	*B. thailandensis*	1.938	*B. pseudomallei*	2.075
10.	2000	*B. pseudomallei* (100)	CP004043.1	*B. thailandensis*	1.934	*B. pseudomallei*	2.063
11.	2001	*B. pseudomallei* (100)	CP004043.1	*B. thailandensis*	1.933	*B. pseudomallei*	2.055
12.	2001	*B. pseudomallei* (100)	CP004043.1	*B. thailandensis*	1.996	*B. pseudomallei*	2.252
13.	2001	*B. pseudomallei* (100)	CP004043.1	*B. thailandensis*	1.808	*B. pseudomallei*	2.017
14.	2001	*B. pseudomallei* (100)	CP004043.1	*B. thailandensis*	1.941	*B. pseudomallei*	2.043
15.	2001	*B. pseudomallei* (100)	CP004043.1	*B. thailandensis*	1.936	*B. pseudomallei*	2.09
16.	2001	*B. pseudomallei* (100)	CP004043.1	*B. thailandensis*	2.016	*B. pseudomallei*	2.077
17.	2001	*B. pseudomallei* (100)	CP004043.1	*B. thailandensis*	1.803	*B. pseudomallei*	2.113
18.	2001	*B. pseudomallei* (100)	CP004043.1	*B. thailandensis*	1.898	*B. pseudomallei*	2.235
19.	2003	*B. pseudomallei* (100)	CP004043.1	*B. thailandensis*	1.946	*B. pseudomallei*	2.27
20.	2003	*B. pseudomallei* (100)	CP004043.1	*B. thailandensis*	1.865	*B. pseudomallei*	2.147
21.	2004	*B. pseudomallei* (100)	CP004043.1	*B. thailandensis*	1.971	*B. pseudomallei*	2.308
22.	2004	*B. pseudomallei* (100)	CP004043.1	*B. thailandensis*	2.002	*B. pseudomallei*	2.303
23.	2005	*B. pseudomallei* (100)	CP004043.1	*B. thailandensis*	1.968	*B. pseudomallei*	2.221
24.	2008	*B. pseudomallei* (100)	CP004043.1	*B. thailandensis*	2.013	*B. pseudomallei*	2.221
25.	2014	*B. pseudomallei* (100)	CP004043.1	*B. thailandensis*	2.013	*B. pseudomallei*	2.176
26.	2014	*B. pseudomallei* (100)	CP004043.1	*B. thailandensis*	2.013	*B. pseudomallei*	2.307

**Table 2:** **Identification of 36 isolates of *B. pseudomallei* obtained from clinical specimens and environmental sources by Bruker Biotyper MALDI-TOF MS with the NTUH-3 strain**.

**No**	**Year of isolation**	**Source**	**Type of infection or environmental sources**	**Identification results by Bruker Biotyper MALDI TOF MS system (NTUH-3 strain)**
27	1994	Human	Mycotic aneurysm (thoracic aorta)	2.545
28	1995	Human	Septic pulmonary emboli	2.375
29	1996	Human	Osteomyelitis, subcutaneous abscess	2.259
30	1996	Human	Pneumonia	2.405
31	1996	Human	Osteomyelitis	2.509
32	1996	Human	Primary bacteremia	2.529
33	1996	Human	Hepatosplenic abscess	2.482
34	1996	Human	Hepatosplenic abscess	2.398
35	1996	Human	Hepatosplenic abscess	2.449
36	1998	Human	Hepatic abscesses	2.567
37	2001	Human	Mycotic aneurysm (thoracic aorta)	2.517
38	2001	Human	Septicemia	2.318
39	2001	Human	Multiple abscesses	2.506
40	2011	Soil	Farm-1 (60 cm below surface)	2.470
41	2011	Soil	Farm-1 (30 cm below surface)	2.626
42	2011	Soil	Lawn (10 cm below surface)	2.508
43	2011	Soil	Lawn (60 cm below surface)	2.436
44	2011	Soil	Lawn (30 cm below surface)	2.461
45	2011	Water	Pond	2.537
46	2012	Soil	Farm-1 (60 cm below surface)	2.425
47	2012	Soil	Farm-1 (60 cm below surface)	2.509
48	2012	Soil	Farm-1 (10 cm below surface)	2.511
49	2012	Soil	Farm-1 (10 cm below surface)	2.315
50	2013	Aerosols	Primary school	2.307
51	2013	Aerosols	Primary school	2.479
52	2013	Aerosols	Primary school	2.385
53	2013	Soil	Farm-1 (60 cm below surface)	2.457
54	2013	Soil	Farm-1 (60 cm below surface)	2.462
55	2013	Soil	Farm-1 (60 cm below surface)	2.413
56	2013	Soil	Farm-1 (60 cm below surface)	2.573
57	2013	Soil	Farm-1 (60 cm below surface)	2.517
58	2013	Soil	Farm-1 (60 cm below surface)	2.509
59	2013	Water	Pond	2.561
60	2013	Water	Pond	2.27
61	2013	Water	Pond	2.436
62	2013	Water	Pond	2.595

*These isolates were identified as B. pseudomallei by sequencing of the 16S rRNA and flagellar genes (6)*.

### Identification of isolates by gene sequencing analysis

Partial 16S rRNA gene sequencing of all 66 isolates was performed using two primers, 8FPL (5′-AGAGTTT GATCCTGGCTCAG-3′) and 1492RPL (5′-GGTTACCTTG TTACGACTT-3′; Cheng et al., 2015). The sequences (1425 bp) obtained were compared with published sequences in the GenBank database using the BLAST algorithm (http://www.ncbi.nlm.nih.gov/blast).

### Identification of isolates by the bruker biotyper MALDI-TOF system

Samples of the 66 isolates were prepared for analysis by the Bruker Biotyper MALDI-TOF MS system as previously described (Cheng et al., 2015). *B. thailandensis* E264 (ATCC700388) was included as the control strain. All isolates were inoculated onto Trypticase soy agar with 5% sheep blood (blood agar plates, BAP; Becton Dickinson Microbiology Systems Sparks, MD, USA) and incubated in 5% CO_2_ at 37°C for 18 to 24 h. Two to three colonies were transferred to a 1.5-ml screw-cap Eppendorf tube containing 50 μl of 70% formic acid. After incubation for 30 s, 50 μl of acetonitrile (Sigma-Aldrich) was added. The suspension was centrifuged at 13,000 rpm for 2 min, and then 1.0 μl of the supernatant was applied to a 96-spot polished steel target plate (Bruker Daltonik GmbH) and dried. A saturated solution of 1.0 μl of MALDI matrix (alphacyano-4-hydroxycinnamic acid matrix solution; Bruker Daltonik GmbH) was applied to each sample and dried. Measurements were performed with the Bruker Biotyper MALDI-TOF MS system using FlexControl software with Compass Flex Series version 1.3 software and a 60-Hz nitrogen laser (337 nm wavelength). Spectra ranging from 2000 to 20,000 *m/z* were analyzed using the MALDI Biotyper system's automation control and the current Bruker Biotyper 3.1 software and library (database [DB] 5627 with 5627 entries). Identification scores of ≥2.000 indicated species-level identification, scores of 1.700 to 1.999 indicated genus-level identification, and scores of < 1.700 indicated no reliable identification. *B. pseudomallei* is not listed in the current Bruker Biotyper MALDI-TOF MS database.

### Cluster analysis and main spectra projection by the Bruker biotyper MALDI TOF system

A clustering analysis of 26 isolates collected from NTUH was performed using ClinProTools 3.0 (Bruker Daltonics GmbH, Bremen, Germany; Cheng et al., 2015). Dendrograms from the MALDI Biotyper data of 26 genetically well-characterized *B. pseudomallei* isolates were obtained and cluster groups with a default critical distance level of 850 were identified. Isolates from different cluster groups were selected for main spectra projection (MSP; database entrance) creation using MALDI Biotyper software (Bruker Daltonics). The database generated using the isolates selected from different cluster groups was blindly tested against the 26 *B. pseudomallei* isolates from NTUH.

External validation was performed for another 36 *B. pseudomallei* isolates collected from southern Taiwan, 30 isolates of *B. putida* collected from NTUH (*n* = 10) and PUMCH (*n* = 20), and 30 isolates of *B. cepacia* complex (from NTUH). Principal component analysis (PCA) dendrograms were generated from MALDI-TOF MS Biotyper mass spectra of 62 isolates of *B. pseudomallei*, including 42 clinical isolates and 20 environmental isolates.

## Results

Among the 30 clinical isolates collected from clinical microbiology laboratories at the NTUH and PUMCH that were initially identified as *B. pseudomallei* (all >90% probability) by the Vietk 2 ID-GN card, four (one for NTUH and three from PUMCH) were identified as non-*B. pseudomallei* by partial 16S rDNA sequencing analysis. Among these four isolates, one was identified as *B. cepacia* complex (NTUH) and three were identified as *B. pudita* (PUMCH) by partial 16S rDNA sequencing analysis.

**Figure 1:**
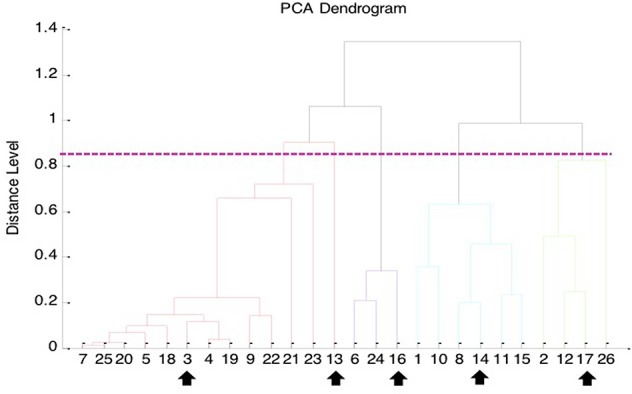
**Principal component analysis (PCA) dendrogram generated from Bruker Biotyper MALDI-TOF MS mass spectra of 26 clinical isolates of ***B. pseudomallei*** collected from NTUH**. The five arrows indicate the MALDI spectra of the five *B. pseudomallei* isolates selected for MSP (main spectra projection; database entrance) creation using MALDI Biotyper software (Bruker Daltonics).

The Bruker Biotyper MALDI-TOF MS system misidentified all the 62 isolates of genetically confirmed *B. pseudomallei* as *B. thailandensis* (seven isolates with a score value ≥2.000) or *Burkholderia* species (19 with score values ranging from 1.803 to 1.996). However, arabinose assimilation testing showed that all 62 isolates were arabinose non-assimilators, indicating that these isolates were not *B. thailandensis*. Clustering analysis of the 26 genetically confirmed isolates of *B. pseudomallei* collected from NTUH by the Bruker Biotyper MALDI-TOF MS system identified five cluster groups (Figure 1).

**Figure 2:**
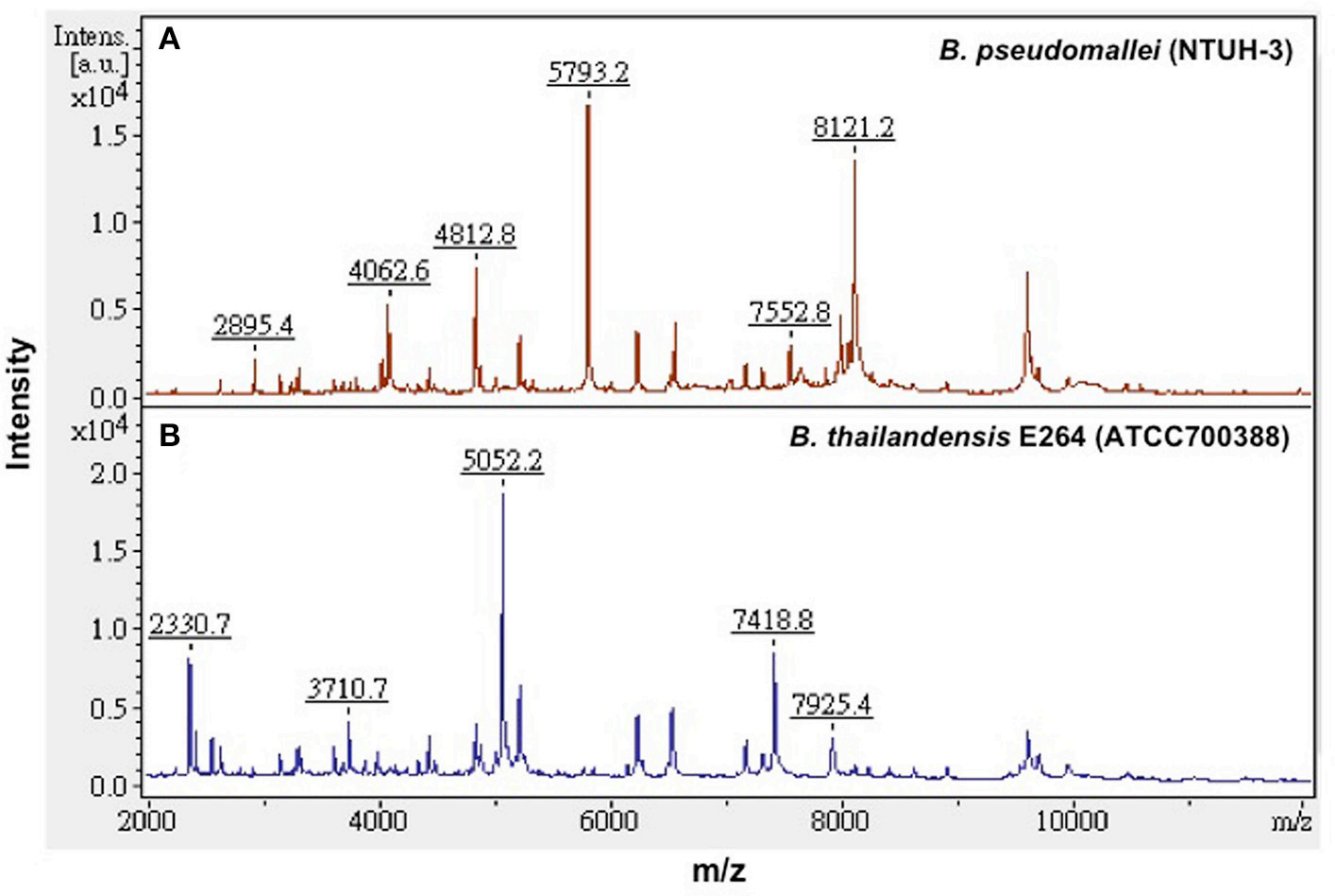
**Characteristic MALDI-TOF MS spectra**. Characteristic spectra of **(A)** *B. pseudomallei* (NTUH-3 strain) and **(B)** *B. thailandensis* E264 (ATCC 700388) generated by the Bruker Biotyper MALDI-TOF MS system. The absolute intensities of the ions are shown on the *y* axis, and the masses (*m/z*) of the ions are shown on the *x* axis. The *m/z* values represent the mass-to-charge ratios.

Because *B. pseudomallei* was not listed in the current Bruker Biotyper MALDI-TOF MS database (DB5627), spectra of five isolates, namely NTUH-3, NTUH-13, NTUH-14, NTUH-16, and NTUH-17, were selected from each cluster group for MSP (database entrance). The database generated using the five isolates was blindly tested against the spectra of the remaining 57 isolates (21 from NTUH and 36 from southern Taiwan). The best identification scores were found according to the database created using the NTUH-3 strain; all (100%) 57 *B. pseudomallei* isolates were correctly identified as *B. pseudomallei* with identification scores of ≥2.000 (2.017 to 2.626). *B. thailandensis* E264 (ATCC700388) was identified by the Bruker Biotyper MALDI-TOF MS system based on Bruker Biotyper 3.1 software and library (DB5627) plus new database created by NTUH-3 strain (enhanced database) as *B. thailandensis* (best match, score value of 2.170) or *B. pseudomallei* (second match, score value of 2.058). Among the four isolates initially mis-identified as *B. pseudomallei*, one was identified as *B. cepacia* complex (NTUH; score value of 2.137) and three were identified as *B. pudita* (PUMCH; 2.012, 2.102, and 2.223, respectively) by standard and enhanced Bruker Biotyper MALDI-TPOF MS database. The characteristic spectra of NTUH-3 strain and *B. thailandensis* E264 (ATCC700388) are shown in Figure 2.

**Figure 3:**
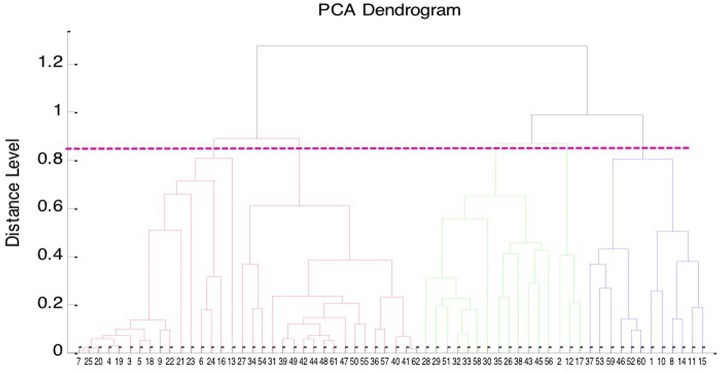
**Principal component analysis (PCA) dendrogram of ***B. pseudomallei*****. PCA dendrogram generated from Bruker Biotyper MALDI TOF MS mass spectra of the 62 isolates of *B. pseudomallei*, including 42 clinical isolates, and 20 environmental isolates collected from northern and southern Taiwan.

The PCA dendrogram generated from MALDI Biotyper mass spectra of 62 isolates of *B. pseudomallei*, including 42 clinical isolates and 20 environmental isolates collected from northern and southern Taiwan is illustrated in Figure 3. There were five cluster groups with dividing branches linked at a distance level of 850.

Among the 30 isolates of genotypically characterized *B. putida* and 30 isolates of *B. cepacia* complex, all were identified as *B. putida* (score values of >2.000) and *B. cepacia* complex (26 *B. cenocepacia* and four *B. cepacia*; score values of >2.000), respectively, using the pre-existing database and that created by NTUH-3 strain.

## Discussion

This study revealed several important findings. First, the Bruker Biotyper MALDI-TOF MS system failed to correctly identify clinical and environmental isolates of *B. pseudomallei* because *B. pseudomallei* is not listed in the current version of FDA-cleared Bruker library. Although there is a “security-relevant library” which contains *B. pseudomallei*, this library is not available in most clinical microbiological laboratories. Second, when we included the *B. pseudomallei* NTUH-3 strain only into the current library, all isolates of *B. pseudomallei* could be identified *B. pseudomallei* by the Bruker Biotyper MALDI-TOF MS system with a correct identification rate of 100%. Third, the control strain of *B. thailandensis* was still identified as *B. thailandensis* as the best match organism when the enhanced database was used. Finally, all isolates obtained from environmental sources were confirmed to be *B. pseudomallei* not *B. thailandensis*.

Identification of *B. pseudomallei* poses difficulties in clinical microbiology laboratories. Although, *B. pseudomallei* is included in the API 20NE, the Vitek 1, and the Vitek 2 databases, the accuracy of identification by these systems varies (Lau et al., 2015). Misidentification of *B. pseudomallei* as other *Burkholderia* species such as *B. cepacia* complex and *B. putida* as well as *Pseudomonas aeruginosa* is common using these commercial systems (around 20%; Lowe et al., 2006; Deepak et al., 2008; Zong et al., 2012; Lau et al., 2015). Furthermore, commercial bacterial identification kits might fail to differentiate between *B. pseudomallei* and a closely related species such as *B. thailandensis*, although >99% of cases of melioidosis are caused by *B. pseudomallei* (Lau et al., 2015). The issue of misidentification of *B. pseudomallei* is of importance for patient care as well as laboratory safety.

Traditionally, *B. pseudomallei* can be distinguished from *B. thailandensis* by arabinose assimilation (Lau et al., 2015). Genotypic differentiation between *B. pseudomallei* and *B. thailandensis* can be achieved by specific PCR-based identification using *B. pseudomallei*-specific gene targets, such as the Type III secretion system and Tat-domain protein and sequencing of gene targets of 16S rRNA and *gro*EL (Lau et al., 2015).

MALDI-TOF MS, a revolutionary technique for pathogen identification, has been shown to be a potentially useful tool for rapid identification of *B. pseudomallei*, although existing databases require optimization by adding reference spectra for *B. pseudomallei* (Inglis et al., 2012; Lau et al., 2012; Niyompanich et al., 2014; Lasch et al., 2015). Jang et al. reported a case of multifocal aneurysms in the aortic arch of the thoracic aorta and pseudoaneurysm in the abdominal aorta and the inferior area of the superior mesenteric artery caused by *B. pseudomallei* (Jang et al., 2015). Colonies from positive blood and tissue were compatible with the presumptive identification of *B. pseudomallei*. However, using the Vitek 2 system, the blood isolate was identified as *B. pseudomallei* and the tissue isolate was identified as *B. cepacia* complex. The blood isolate was identified as *B. thailandensis* with a score value of 1.901 by the Bruker Biotyper MALDI-TOF MS system (Jang et al., 2015). Lau et al. used the Bruker database extended with *B. pseudomallei* reference strains, three *B. thailandensis* isolates were misidentified as *B. pseudomallei*. In this study, the reference strain of *B. thailandensis* was correctly identified with enhanced Bruker database. More isolates of *B. thailandensis* isolates are needed for verify the accuracy of this new database.

Recently, bioMerieux recognized misidentification of *B. pseudomallei* as an important issue and addressed the problem by altering the algorithm parameters. The new parameters are included in the most recent software release (version 4.03 for Vitek 2 60/XL and version 2.01 for Vitek 2 Compact; Lowe et al., 2006).

Interestingly, in this study all four isolates initially identified as *B. pseudomallei* by the Vitek 2 ID-GN card were identified as *B. cepacia* complex or *B. pudita* by 16S rRNA sequencing analysis. In areas with high endemicity of melioidoisis like Taiwan, laboratory staff tends to report the results of *B. pseudomallei* isolation without further clarification. In contrast, most clinical microbiologists in Beijing often recheck the identification of *B. pseudomallei* because of the low incidence of melioidoisis in northern China (Yang, 2000; Currie et al., 2008; Fang et al., 2015; Zheng et al., 2015).

## Summary

In areas where this organism is endemic, such as in South Asia and Northern Australia, identification of *B. pseudomallei* is not usually problematic (Currie et al., 2008; Lau et al., 2015). With increased international travel and threats of bioterrorism, recognition, and accurate identification of these organisms is important (Lau et al., 2015). The use of automated identification systems, including MALDI-TOF MS, in the clinical microbiology laboratory is becoming common as the pressure of cost containment impacts staff resources. In this study, using our newly created database, all *B. pseudomallei* isolates were correctly identified to the species level using the Bruker Biotyper MALDI-TOF MS system. These findings suggest that MALDI-TOF MS is a versatile and robust tool for the rapid identification of *B. pseudomallei* isolates. Expansion of commercially available databases with pathogens endemic in different regions is crucial to improve the usefulness of MALDI-TOF MS. However, this successful application of MALDI-TOF can only be regarded as pilot study, due to the small sample size, which needs independent validation before it can be offered as routine technique in the clinic.

## Author contributions

HW, PH, and YX conceived and designed the experiments, performed the experiments, analyzed the data, and wrote the paper. YC, ST, ZX performed the experiments and analyzed the data. HW, YC, ST, ZX, PH, YX read and approved the final version of the manuscript.

### Conflict of interest statement

The authors declare that the research was conducted in the absence of any commercial or financial relationships that could be construed as a potential conflict of interest.
